# Activity and stability studies of H-transfer reduction reactions of aldehydes and ketones over aluminium isopropoxide heterogenised catalysts†

**DOI:** 10.1039/d2ra06437e

**Published:** 2022-11-29

**Authors:** Atika Muhammad, Ammaru Ismaila, Bashir Jelani Usman, Graziano Di Carmine, Carmine D'Agostino

**Affiliations:** Department of Chemical Engineering, The University of Manchester Oxford Road M13 9PL UK carmine.dagostino@manchester.ac.uk; Department of Materials, The University of Manchester Oxford Road M13 9PL Manchester UK; Dipartimento di Scienze Chimiche, Farmaceutiche ed Agrarie, Università degli Studi di Ferrara Via L. Borsari, 46 I-44121 Ferrara Italy; Dipartimento di Ingegneria Civile, Chimica, Ambientale e dei Materiali (DICAM), Alma Mater Studiorum – Università di Bologna Via Terracini, 28 40131 Bologna Italy carmine.dagostino@unibo.it

## Abstract

Aluminium isopropoxide Al(O^i^Pr)_3_ immobilised on various mesoporous supports (SiO_2_, TiO_2_ and γ-Al_2_O_3_) was tested for H-transfer reductions of various aldehydes and ketones in the presence of 2-propanol as a sacrificial agent. The heterogenised catalysts were characterised by N_2_ physisorption, XRD, SEM-EDX, FTIR and ICP-OES. The characterisation results show a successful grafting of the homogeneous aluminium isopropoxide catalyst, covalently bound to the solid surface, with high dispersion over the mesoporous supports. The heterogenised catalysts show an excellent catalytic activity with high selectivity towards the desired alcohol product, with performances that are comparable with those of the homogeneous Al(O^i^Pr)_3_ catalyst. Al(O^i^Pr)_3_ grafted on SiO_2_ shows higher activity compared to γ-Al_2_O_3_ and TiO_2_ supported catalysts. The catalysts remain very active after 5 cycles of reuse and no leached Al(O^i^Pr)_3_ was found in the reaction mixture, hence showing an excellent stability. The work reported here shows that it is possible to effectively immobilise catalytic functions, usually working in the homogeneous phase, over solid supports, with the resulting heterogenised catalysts keeping the same catalytic activity of the homogeneous counterpart and excellent stability, and with the advantage of being able to recycle and reuse them, without loss of catalytic materials.

## Abstract 2

Aluminium isopropoxide Al(O^i^Pr)_3_ immobilised on various mesoporous supports (SiO_2_, TiO_2_ and γ-Al_2_O_3_) was tested for H-transfer reductions of various aldehydes and ketones exhibiting excellent activity and stability.

## Graphical abstract

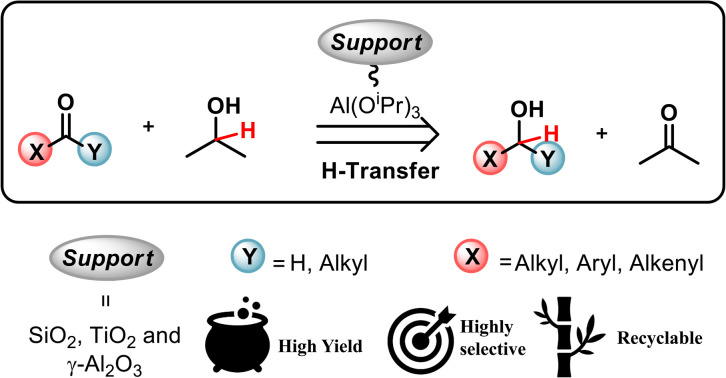

## 1 Introduction

H-transfer reduction of aldehydes and ketones through the Meerwein–Ponndorf–Verley (MPV) reaction is a well-known route for the production of the corresponding alcohols. Typically, a secondary alcohol, such as 2-propanol, serves as the hydride donor. The reaction involves a hydride transfer from a secondary alcohol to a carbonyl substrate *via* a six-membered transition state, initiated by the activation of the carbonyl groups by coordination to the Lewis acidic aluminium centre.^1,2^ In unsaturated aldehydes and ketones, the double bond is untouched and no saturated counterparts are observed during the MPV reduction, which makes the reaction chemoselective towards the reduction of the carbonyl group only, which is a key advantage of such reactions. As a result, the MPV reduction provides a practical method for synthesising unsaturated alcohols, many of which are crucial raw materials for the synthesis of fine chemicals.

Traditionally, aluminium alkoxides, such as aluminium isopropoxide and other aluminium complexes, have been used to homogeneously catalyse the reaction.^3–6^ Aluminium precatalysts derived from alkyl aluminium complexes have shown high activity for MPV reduction of aldehydes and ketones in toluene.^7^ In addition to aluminium alkoxide complexes, other metal complexes, such as lanthanum alkoxides,^8^ have been reported to be active catalysts for MPV reduction of carbonyl compounds. However, most of these catalysts are homogeneous and are therefore difficult to separate from the reaction mixture, which makes their reuse challenging, if not unfeasible. In this context, the use of solid catalysts would certainly be advantageous from a practical perspective; hence the search for reusable solid catalysts, with similar effectiveness as the homogeneous ones, has often been pursued in recent decades. Lopez *et al.* reported the reduction of 4-*tert*-butylcyclohexanone using mixed oxides obtained from hydrotalcites, NaBEA zeolites, KF/alumina and La_2_O_3_.^9^ The findings suggest that basic sites are the catalyst active sites.^9^ Other solid catalysts such as BEA zeolites,^10^ magnesium phosphates^11,12^ and zirconia^13^ have been previously reported. However, these catalysts suffer from poor selectivity. The use of supported homogenous catalysts has emerged as an alternative to the inadequate separation of homogenous catalysts and the poor selectivity of conventional heterogeneous solid catalysts. Supported metal alkoxides such as zirconium isopropoxide,^14,15^ boron isopropoxide,^16^ indium isopropoxide,^17^ lanthanum alkoxides^9^ and ceric alkoxide^18^ have been reported to be active catalysts for MPV reduction. MCM-41 was employed by Anwander *et al.* as a support material for the grafting of aluminium isopropoxide.^19^ It was discovered that the hybrid system was particularly active in the MPV reduction of 4-*tert*-butylcyclohexanone. The immobilised catalyst demonstrated excellent activity with 88% conversion of 4-*tert*-butylcyclohexanone after 5 hours and >99% conversion after 24 hours. However, the sacrificial alcohol must be thoroughly dried to achieve good catalytic yield.^19^ Despite the application of a range of aluminium alkoxide catalysts in MPV reduction, the active aluminium species are still poorly understood. In the crystal form of the aluminium isopropoxide catalyst, the six-coordinated aluminium centre is surrounded by three bridging Al(O^i^Pr)_3_ groups, but in solution a variety of species forms and their simple interconversions makes it difficult to predict the active aluminium sites.^20^ As previously mentioned, the use of homogeneous catalysts has also some drawbacks in terms of catalyst separation, reuse and recycling, which is often unfeasible. In order to overcome these practical issues associated with homogeneous catalysis, the heterogenisation of aluminium isopropoxide over solid supports is appealing since it makes the catalytic function insoluble, easy to recycle and reuse, with the possibility to be engineered also in continuous fixed-bed reactors.^21,22^ Whilst SiO_2_-based materials have previously been investigated as potential supports,^19,23^ the use of other types of supports has remained largely unexplored.

In this study, we investigate H-transfer reduction reactions of carbonyl compounds (aldehydes and ketones) catalysed by aluminium isopropoxide immobilised over mesoporous solid supports. The use of high surface area supports for the immobilisation is expected to enhance the dispersion of the aluminium isopropoxide catalyst. The influence of the type of support studied, namely SiO_2_, TiO_2_ and γ-Al_2_O_3_, was investigated. Several aldehydes and ketones were used in the reaction screening and the results on catalytic performances are compared with those of the same aluminium isopropoxide catalyst in homogeneous solution.

## 2 Experimental

### 2.1. Materials and chemicals

Aluminium isopropoxide (98%), 2-propanol (anhydrous, 99.5%), acetophenone (99%), cyclohexanone (≥99.5%), benzaldehyde (≥99%), *trans*-cinnamaldehyde (99%), *n*-hexane (anhydrous, >99%), silica (SiO_2_) and titanium(iv) oxide (TiO_2_), anatase phase, were obtained from Sigma Aldrich, UK. Propionaldehyde (extra pure, SLR) was obtained from Fisher Scientific UK, while aluminium oxide (Al_2_O_3_), γ-phase, from Alfa Aesar.

### 2.2. Preparation of supported catalysts

Aluminium isopropoxide Al(O^i^Pr)_3_ was grafted onto the various supports according to a method previously published in the literature.^24^ The support (SiO_2_, TiO_2_, γ-Al_2_O_3_) was dried for 4 hours at a temperature of 250 °C prior to grafting. The heterogenised homogeneous catalyst was made by mixing 5 mmol of aluminium isopropoxide in 25 ml of dry *n*-hexane with 2 g of the support (SiO_2_, TiO_2_, γ-Al_2_O_3_). The mixture was refluxed at 69 °C for 12 hours while being agitated at 500 to 700 rpm. The suspension was filtered in a N_2_ atmosphere, washed with *n*-hexane three times, and dried in an inert environment. Al(O^i^Pr)_3_-SiO_2_, Al(O^i^Pr)_3_-TiO_2_ and Al(O^i^Pr)_3_-Al_2_O_3_ final products have 2.45 mmol, 2.23 mmol and 2.39 mmol Al per gram of catalyst, respectively.

### 2.3. Characterisation of materials

The surface area of the supports and grafted catalysts were analysed using a Micromeritics surface area analyser. Testing vials were first heated at 100 °C for 2 hours and purged under N_2_ gas. All samples were heated to 300 °C under vacuum for 6 hours. About 0.1–0.2 g of sample was used. Nitrogen adsorption and desorption isotherms were recorded at liquid nitrogen temperature of −196 °C. Specific surface area was calculated using Brunauer–Emmett–Teller (BET) method while pore size analysis was conducted by the Barrett–Joyner–Halenda (BJH) method using the adsorption branch. The crystalline phases of the support and prepared catalyst were analysed using X-ray diffraction measurements. The XRD patterns were collected using Philips X'Pert Xray diffractometer operated at 40 kV and 40 mA with a Cukα1 X-ray source (*λ* = 0.154 6 nm) in a 2*θ* range of 20° to 80° with 0.02° step size. To identify the relevant phases, the XRD pattern were analysed using JADE 6 (Material Data Inc., Livermore, CA) to compare with standard structures in the International Centre for Diffraction Data (ICDD) database. Surface morphology and EDX analysis of the samples was performed using Quanta 250. Samples were prepared by first dissolving in ethanol and sprinkled onto carbon tape stuck to an aluminium stub. To make the samples conductive, the samples were dried under light and coated with platinum using Cressington Platinum (Pt) Sputter Coater for about 50 s (approximately 10 nm thickness). The actual quantity of aluminium accessible in the catalysts was determined using inductively coupled plasma optical emission spectrometry (ICP-OES, Plasma Quant PQ 9000). Prior to ICP analysis, the catalysts were typically microwave-digested in acid solution (HCl, H_2_SO_4_ and HNO_3_). An acid solution containing aluminium was used as standard reference. The FTIR spectra of the samples were collected using a Bruker Vertex 7.0 Fourier transform infrared (FTIR) spectrometer with a scanning wavenumber (ranging from 400 to 4000 cm^−1^) and a spectral resolution of 4 cm^−1^.

### 2.4. Reaction studies

In a 50 ml round bottom flask equipped with reflux condenser, thermometer and a magnetic stirrer, the heterogenised catalyst (200–220 mg) was added to the reaction mixture containing 1.4 mmol of the carbonyl compound of interest and 60 mmol of 2-propanol. Anhydrous 2-propanol was used as previous studies suggest that drying of the 2-propanol could significantly improve catalytic activity of aluminium isopropoxide catalyst in MPV reduction.^19^ An excess of 2-propanol was also necessary to shift the equilibrium reaction to the desired product. The mixture was stirred at 750 rpm and heated to reflux. The mixture was analysed using Agilent 7820A gas chromatography system equipped with FID detector and a HP-5 methylpolysiloxane column (30 m × 320 μm × 0.25 μm). Products were identified by their retention time and compared with authentic samples. Yield was calculated using eqn (S1) of the ESI.†

At the end of each round of reaction, the spent catalyst was recovered by filtration. The reuse of the catalyst was tested choosing the reduction of propionaldehyde as benchmark reaction. After the reaction, the spent catalysts were washed several times with 2-propanol, dried at 80 °C for 6 hours and subsequently tested again for the reduction of fresh propionaldehyde through 5 rounds of reaction under the same conditions.

To assess for leaching, 200 mg of the heterogenised catalyst, Al(O^i^Pr)_3_-SiO_2_, Al(O^i^Pr)_3_-TiO_2_ and Al(O^i^Pr)_3_-Al_2_O_3_, was refluxed in 60 mmol 2-propanol at 80 °C for 4 hours. The solution was filtered, and the filtrate was evaluated for reduction of propionaldehyde.

## 3 Results and discussion

### 3.1. Characterisation of supports and heterogenised catalysts

**Fig. 1:**
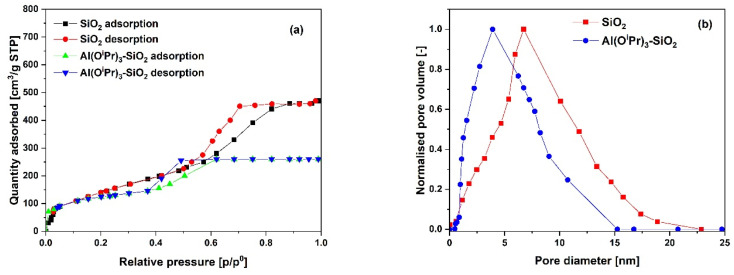
(a) N_2_ adsorption–desorption isotherms and (b) pore size distribution (PSD) of the SiO_2_ support and Al(O^i^Pr)_3_-SiO_2_ catalyst.

**Fig. 2:**
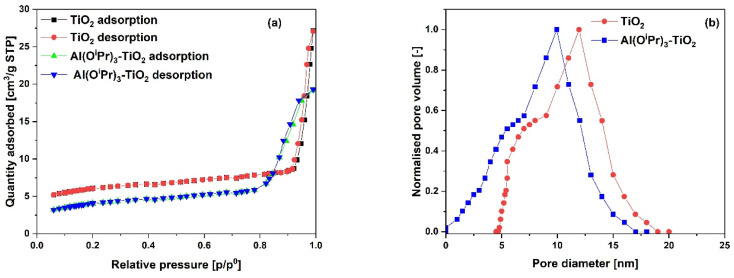
(a) N_2_ adsorption–desorption isotherms and (b) pore size distribution (PSD) of the TiO_2_ support and Al(O^i^Pr)_3_-TiO_2_ catalyst.

**Fig. 3:**
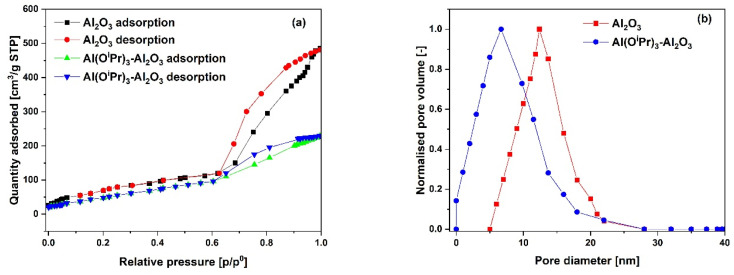
(a) N_2_ adsorption–desorption isotherms and (b) pore size distribution (PSD) of the γ-Al_2_O_3_ support and Al(O^i^Pr)_3_-Al_2_O_3_ catalyst.

The N_2_ adsorption–desorption isotherms for the SiO_2_ support and Al(O^i^Pr)_3_-SiO_2_ with corresponding pore size distribution (PSD) are shown in Fig. 1(a) and (b), respectively, while for γ-Al_2_O_3_ support and Al(O^i^Pr)_3_-Al_2_O_3_ are shown in Fig. 3(a) and (b), respectively. Both the support and catalyst (SiO_2_, Al(O^i^Pr)_3_-SiO_2_; γ-Al_2_O_3_, Al(O^i^Pr)_3_-Al_2_O_3_) show a type IV isotherm. The adsorption isotherm can be categorised into three parts: the monolayer multiple adsorptions of N_2_ on the surface of the mesoporous material, capillary condensation of the N_2_ into the mesopores and then saturation. The adsorption and desorption isotherms for both samples are not superimposed. A phenomenon referred to as hysteresis, which is linked to capillary condensation occurring in the mesopores, is an indication that the materials are mesoporous. The capillary condensation for untreated SiO_2_ occurs at relative pressure *p*/*p*° = 0.50–0.82. After grafting with aluminium isopropoxide, the capillary condensation is observed to shifts towards lower region of the relative pressure. This suggests changes in the mesoporous structure of the support after incorporation with the homogeneous aluminium isopropoxide catalyst as suggested by the data in Table 1. As expected, the grafting of aluminium isopropoxide decreases the surface area of the SiO_2_ and γ-Al_2_O_3_ supports as well as the pore volume and pore diameter (Table 1). The Brunauer–Emmett–Teller (BET) surface area, *S*_BET_ and pore volume, *V*_p_ of Al(O^i^Pr)_3_-SiO_2_ (*S*_BET_ = 408 m^2^ g^−1^ and *V*_p_ = 0.4 cm^3^ g^−1^) are slightly lower than those of SiO_2_ (*S*_BET_ = 434 m^2^ g^−1^ and *V*_p_ = 0.73 cm^3^ g^−1^). Similarly, the *S*_BET_ and *V*_p_ of Al(O^i^Pr)_3_-Al_2_O_3_ (*S*_BET_ = 212 m^2^ g^−1^ and *V*_p_ = 0.35 cm^3^ g^−1^) are lower than those of γ-Al_2_O_3_ (*S*_BET_ = 240 m^2^ g^−1^ and *V*_p_ = 0.74 cm^3^ g^−1^). The N_2_ adsorption–desorption isotherms for the TiO_2_ support and Al(O^i^Pr)_3_-TiO_2_ with the corresponding pore size distribution (PSD) are shown in Fig. 2(a) and (b), respectively. TiO_2_ and Al(O^i^Pr)_3_-TiO_2_ show a type III isotherm, an indication that the material is mesoporous with weak adsorbate–adsorbent interaction.^25^ The grafting of aluminium isopropoxide again decrease the surface area of the TiO_2_ support as well as the pore volume and pore diameter (Table 1), but the material still retains its characteristics of ordered mesoporous material. The observed decrease in surface area, pore volume and pore diameter may be due to plugging of several pores of the mesoporous material by the aluminium isopropoxide catalyst.^23^

**Table 1:** Textural properties of the mesoporous supports and prepared catalysts

Sample	*S*_BET_ a (m^2^ g^−1^)	*V*_p_ b (cm^3^ g^−1^)	*D*_p_ (nm)	Al^c^ (wt%)
SiO_2_	434	0.73	6.73	—
Al(O^i^Pr)_3_-SiO_2_	408	0.40	3.92	6.61
TiO_2_	14	0.04	11.96	—
Al(O^i^Pr)_3_-TiO_2_	12	0.03	9.94	5.99
γ-Al_2_O_3_	240	0.74	12.39	—
Al(O^i^Pr)_3_-Al_2_O_3_	212	0.35	6.68	6.45

a Determined by BET method.

b Calculated from volume of N_2_ adsorbed at *p*/*p*° = 0.99.

c Determined by ICP-OES.

ICP-OES analysis was used to assess how much aluminium was grafted across the three support samples. The Al content over the silica and alumina support was found to be almost similar. The result show 6.45 wt% aluminium content for the alumina support while Al(O^i^Pr)_3_-SiO_2_ shows a slightly higher content of 6.61 wt%. The slightly lower percentage of Al found in the Al(O^i^Pr)_3_-Al_2_O_3_ sample can be attributed to limited accessibility to the surface hydroxyl groups in Al(O^i^Pr)_3_ or due to variation in physicochemical properties between the two mesoporous supports (Table 1). Al(O^i^Pr)_3_-TiO_2_ has the lowest Al content, 5.99 wt%, in comparison to the other samples. The lower Al loading may be attributed to the smaller surface area and pore volume than those found in Al(O^i^Pr)_3_-SiO_2_ and Al(O^i^Pr)_3_-Al_2_O_3_ catalysts (Table 1). A previous study indicates that smaller surface area could lead to lower catalyst loading by restricting the grafting to not more than a monolayer.^14^ Al(O^i^Pr)_3_-SiO_2_, out of the three catalysts, exhibits the highest Al content, possibly indicating a higher proportion of silanol groups for the stabilisation of the Al species.

**Fig. 4:**
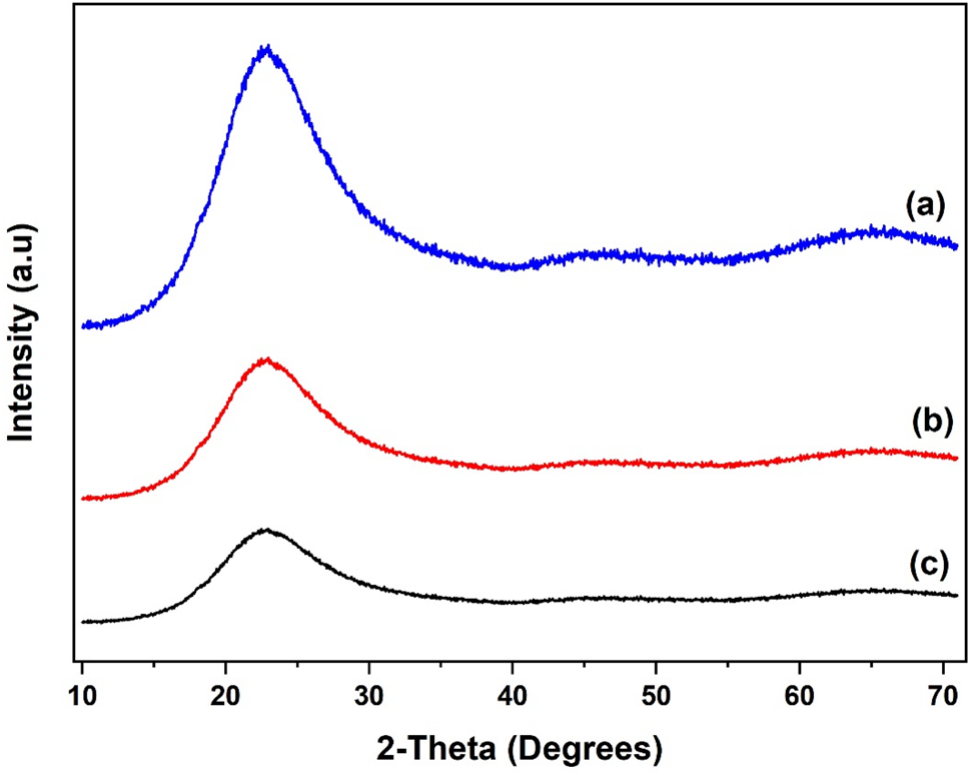
XRD patterns of (a) SiO_2_ support, (b) Al(O^i^Pr)_3_-SiO_2_ and (c) recycled Al(O^i^Pr)_3_-SiO_2_.

**Fig. 5:**
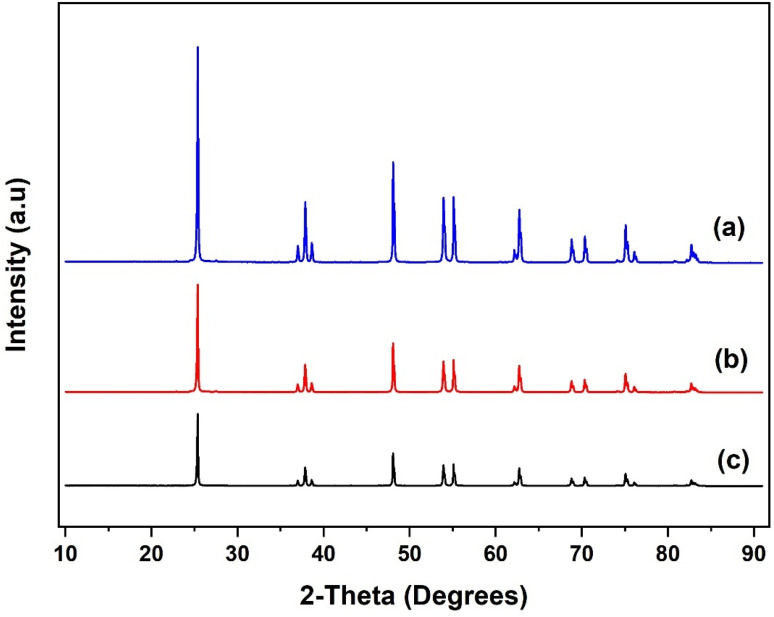
XRD patterns of (a) TiO_2_ support, (b) Al(O^i^Pr)_3_-TiO_2_ and (c) recycled Al(O^i^Pr)_3_-TiO_2_.

**Fig. 6:**
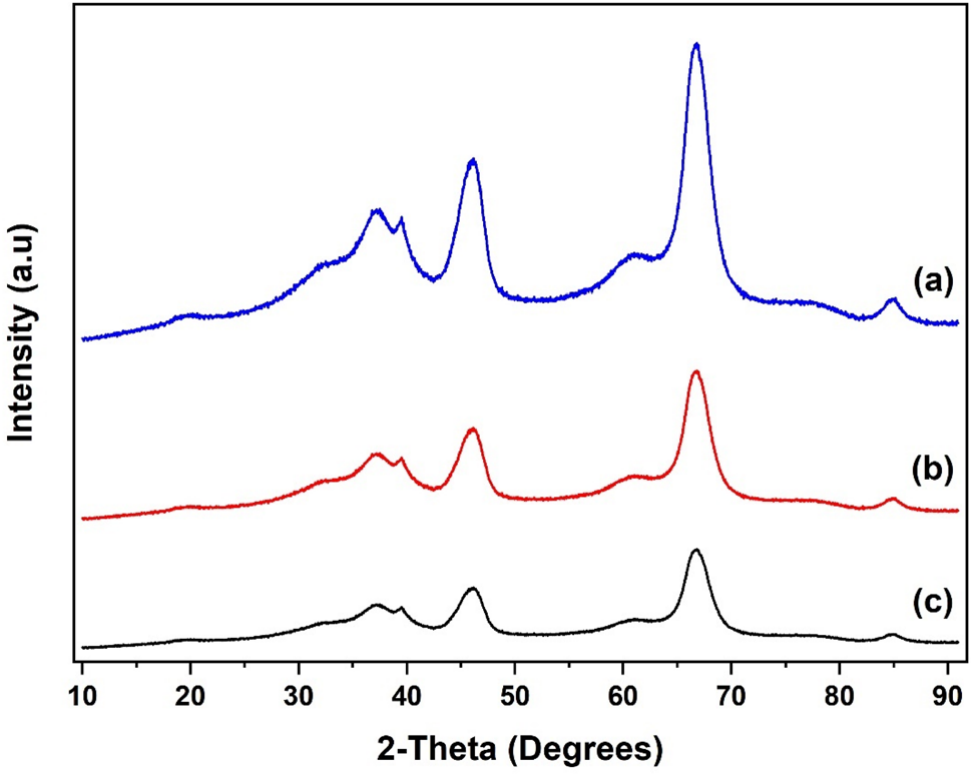
XRD patterns of (a) γ-Al_2_O_3_ support, (b) Al(O^i^Pr)_3_-Al_2_O_3_ and (c) recycled Al(O^i^Pr)_3_-Al_2_O_3_.

The XRD patterns of the SiO_2_ support and the grafted Al(O^i^Pr)_3_-SiO_2_ are presented in Fig. 4. The pattern for pure SiO_2_ support shows a characteristic peak of the amorphous silica broad at approximately 10–30° (Fig. 4(a)). After grafting, Al(O^i^Pr)_3_-SiO_2_ displays similar pattern as the support as shown in Fig. 4(b), which indicates that the homogeneous aluminium isopropoxide catalyst is highly dispersed on the support. The intensity of the peak is also observed to weaken after grafting of the aluminium isopropoxide, which may be related to the integration of aluminium complexes into the channels of the SiO_2_ substrate. Moreover, the observed decrease in intensity is an indication of reduced pore size after grafting, as suggested by the surface area and pore volume size (Table 1). For TiO_2_, value of peaks at (2*θ* = 25.3°, 37.8°, 48.1°, 53.9°, 55.3°, 62.7°, 70.3°, 75.1° and 82.8°) match very closely with those reported in JCPDS file (21-1272). The peaks match with the characteristic peaks of the anatase phase of TiO_2_ with the most intense peak at 25.36°. The diffraction peaks correspond to (101), (004), (200), (105), (211), (204) and (215) orientations (Fig. 5). For Al(O^i^Pr)_3_-TiO_2_, only peaks from TiO_2_ emerge and no peak from aluminium isopropoxide could be detected. This indicates that at appropriate loading, aluminium isopropoxide is highly dispersed on the surface of the TiO_2_ support (Fig. 5(b)). The X-ray diffraction pattern of the γ-Al_2_O_3_ support shows peaks at 2*θ* = 37.3°, 39.5°, 46.5°, 61.1°, 67.1° and 85.2°, which match closely with those on JCPDS file (46-1131), (Fig. 6(a)). Al(O^i^Pr)_3_-Al_2_O_3_ shows a similar pattern to that of the γ-Al_2_O_3_ support as no new peaks were detected (Fig. 6(b)).

**Fig. 7:**
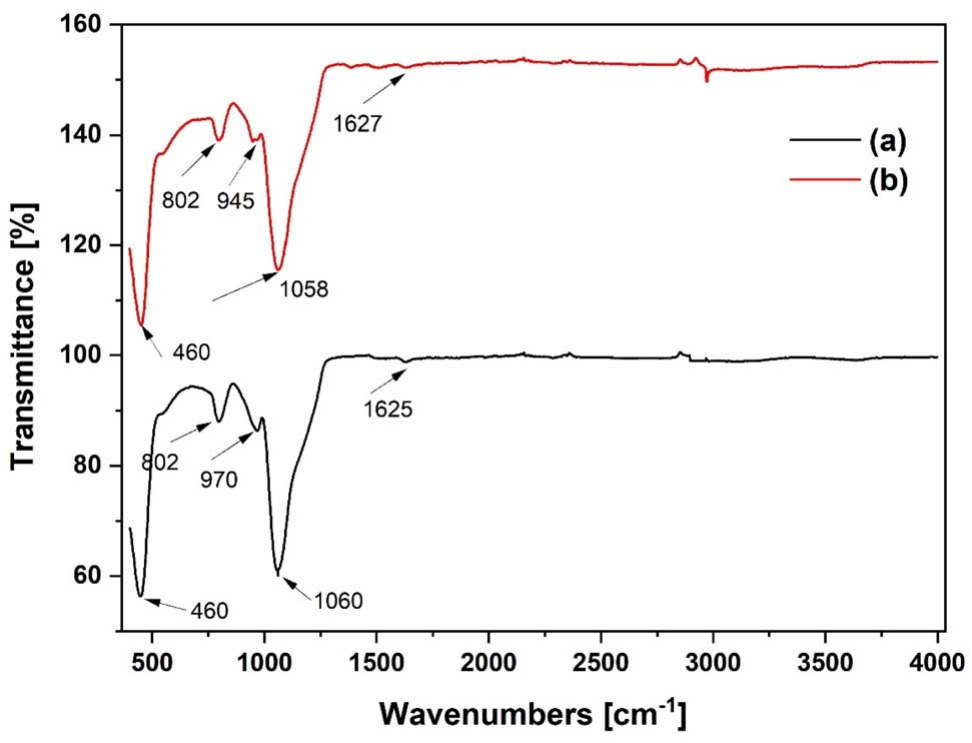
FTIR spectra of (a) pure SiO_2_ and (b) Al(O^i^Pr)_3_-SiO_2_.

Fig. 7 displays the FTIR spectra of the SiO_2_ and Al(O^i^Pr)_3_-SiO_2_. The major characteristic peaks of Si–O–Si vibrations are observed at 460 cm^−1^, 802 cm^−1^ and 1060 cm^−1^.^26^ The peak at 460 cm^−1^ is usually associated with Si–O/Al–O bending vibration whilst the peak at 802 cm^−1^ is assigned to Si–O–Si symmetric stretching vibration.^27^ The strong band at 1060 cm^−1^ is attributed to Si–O–Si/Si–O–Al asymmetric stretching.^28^ The band at 970 cm^−1^ is assigned to Si–OH stretching.^29,30^ Comparing the 970 cm^−1^ peak of the SiO_2_ support to the 945 cm^−1^ peak of the grafted Al(O^i^Pr)_3_-SiO_2_, we found that the latter shifts toward a lower wave number. This may be explained by the formation of Al–O–Si bonds, which result from the interaction of aluminium and silicon atoms.^31^ This also suggests that the Al species are covalently bound to the surface of the SiO_2_ support.

**Fig. 8:**
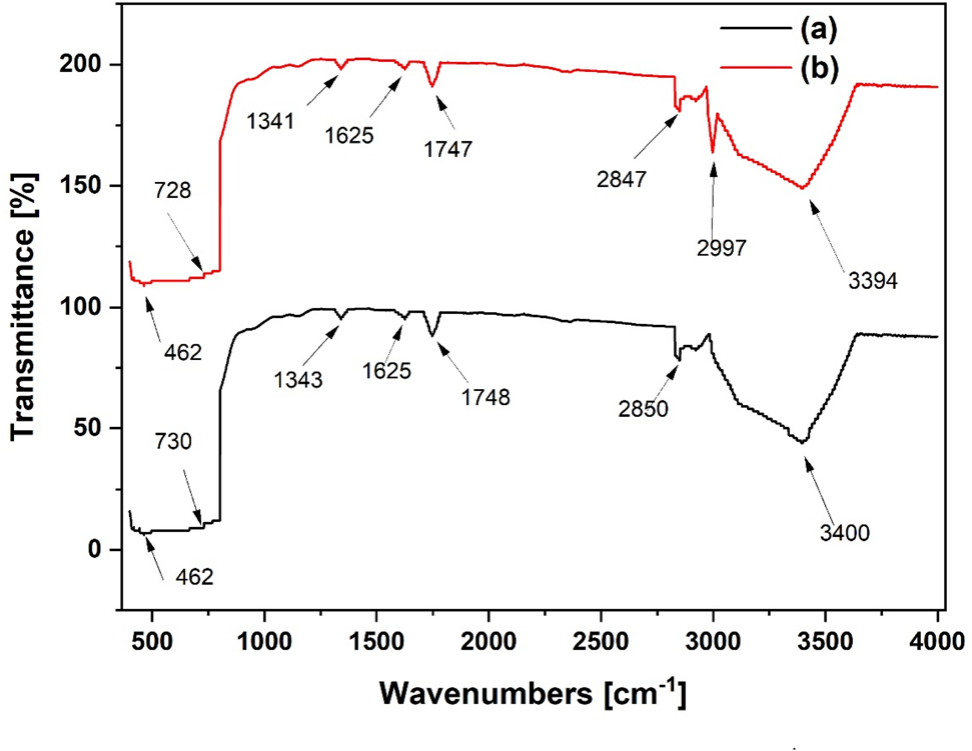
FTIR spectra of (a) pure TiO_2_ (b) and Al(O^i^Pr)_3_-TiO_2_.

The FTIR spectra of TiO_2_ and Al(O^i^Pr)_3_-TiO_2_ materials are shown in Fig. 8. The peak at 462 cm^−1^ in both materials is attributed to the vibration of the Ti–O bond in the TiO_2_ matrix. The peaks observed at 3400 and 3394 cm^−1^ for TiO_2_ and Al(O^i^Pr)_3_-TiO_2_ are attributed to symmetric and asymmetric vibrations of Ti–OH.^32^ The absorption band observed at 730 and 728 cm^−1^ are associated with Ti–O–Ti stretching vibrations. For pure titanium oxide, the contributions from the anatase titania are visible in the range of 400–800 cm^−1^.

**Fig. 9:**
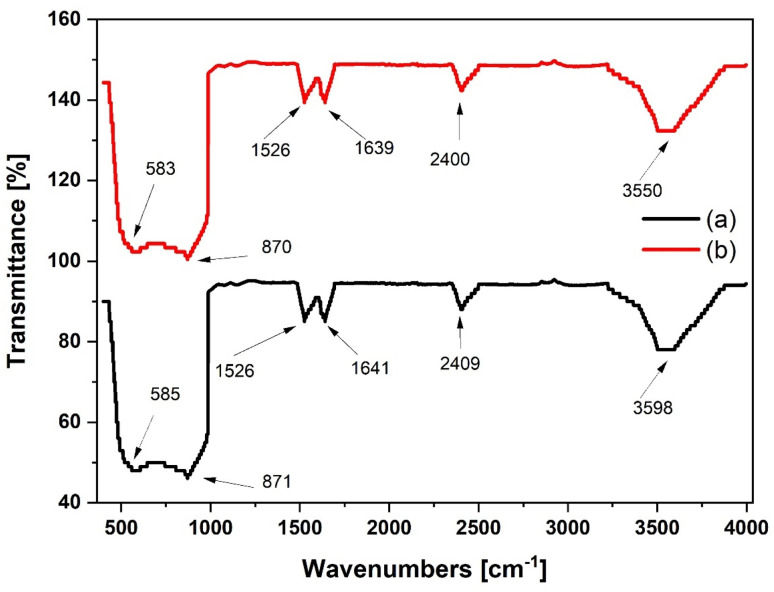
FTIR spectra of (a) pure Al_2_O_3_ and (b) Al(O^i^Pr)_3_-Al_2_O_3_.

The FTIR spectra of the gamma alumina measured between 400 to 4000 cm^−1^ is shown in Fig. 9. The presence of the gamma form is confirmed by the measured peaks for the bending vibrations of Al–O–Al at 585 and 871 cm^−1^ for Al_2_O_3_ and 583 and 870 cm^−1^ for Al(O^i^Pr)_3_-Al_2_O_3_. Intensities of the strongest peaks are observed to increase slightly for Al(O^i^Pr)_3_-Al_2_O_3_. Peaks at 585, 2409 and 398 cm^−1^ for the Al_2_O_3_ support slightly move towards lower region of wavenumbers to 583, 2400 and 3598 cm^−1^ for the grafted Al(O^i^Pr)_3_-Al_2_O_3_. These changes could be due to the grafting of the aluminium isopropoxide catalyst on the support.

**Fig. 10:**
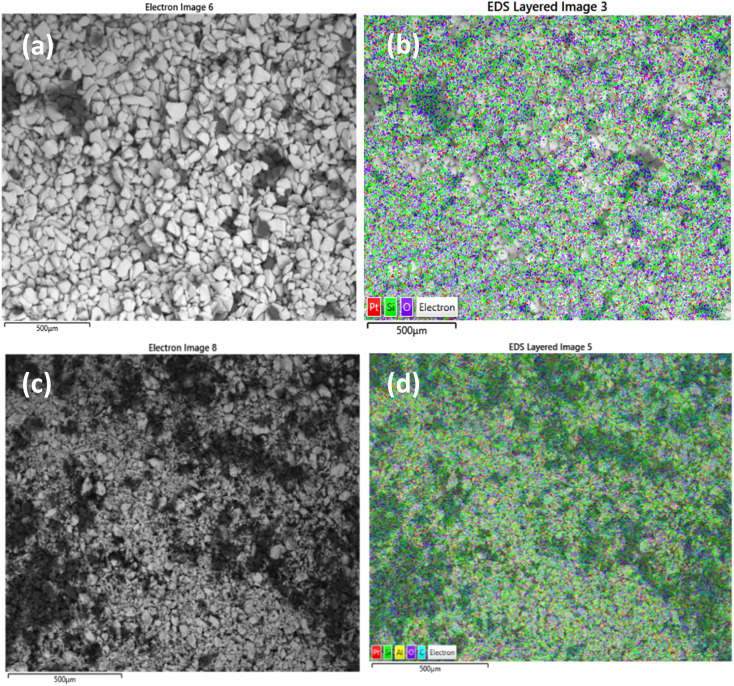
SEM and EDX elemental mapping of (a), (b) SiO_2_ and (c), (d) Al(O^i^Pr)_3_-SiO_2_. Pt comes from the coating of the sample as part of sample preparation for the analysis.

**Fig. 11:**
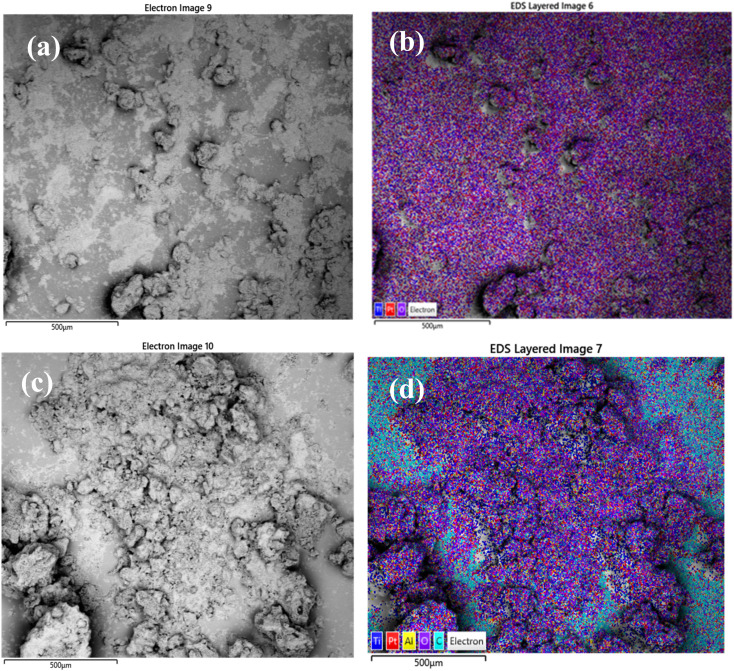
SEM and EDX elemental mapping of (a), (b) TiO_2_ and (c), (d) Al(O^i^Pr)_3_-TiO_2_. Pt comes from the coating of the sample as part of sample preparation for the analysis.

**Fig. 12:**
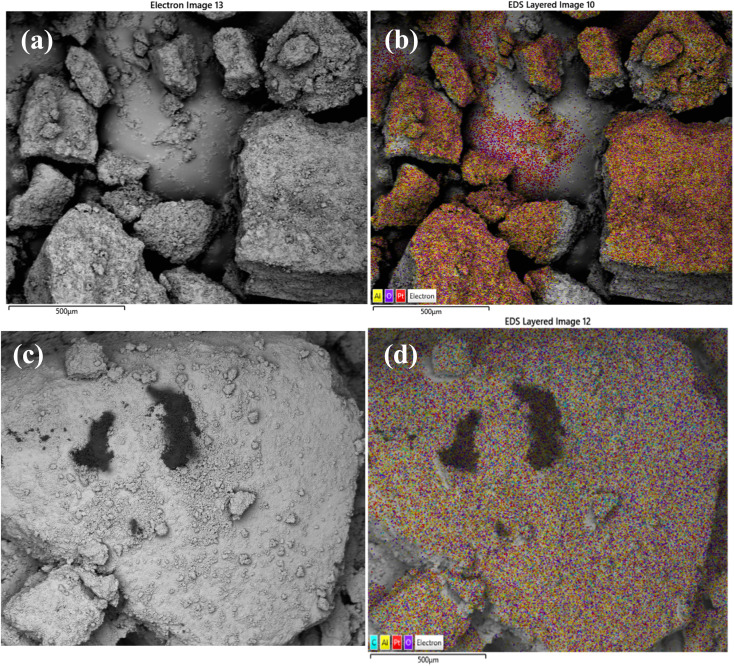
SEM and EDX elemental mapping of (a), (b) γ-Al_2_O_3_ and (c), (d) Al(O^i^Pr)_3_-Al_2_O_3_. Pt comes from the coating of the sample as part of sample preparation for the analysis.

Scanning electron microscopy was used to examine morphology and size distribution of the support and grafted catalyst. Elemental dispersive X-ray spectroscopy (EDX-SEM) was used to examine the elemental distribution and content of the materials. SEM images and elemental distributions of the supports and grafted catalysts are shown in Fig. 10, 11 and 12. This analysis confirms the presence of aluminium isopropoxide on the silica, titania and alumina supports. When compared to EDX spectra of pure SiO_2_, TiO_2_ and Al_2_O_3_ (Fig. S1(a), S2(a) and S3(a) of the ESI†), the spectra of Al(O^i^Pr)_3_-SiO_2,_ Al(O^i^Pr)_3_-TiO_2,_ Al(O^i^Pr)_3_-Al_2_O_3_ clearly showed Al and C signals (Fig. S1(b), S2(b) and S3(b) of the ESI†). This shows unequivocally that aluminium and carbon are included in the constructed heterogeneous catalyst, thus proving the successful grafting of the homogeneous Al(O^i^Pr)_3_. The figures also show homogeneous dispersion of the aluminium isopropoxide over the various supports. This gives compelling evidence that most of the Al species are evenly distributed within or on top of the support materials. Catalyst supports are also free of contaminants within the examined range.

### 3.2. Catalytic H-transfer reaction activity of the heterogenised catalysts

The feasibility of using homogeneous aluminium isopropoxide for H-transfer reduction though MPV reaction of propionaldehyde has been previously reported.^33–35^ In this work we describe the preparation and characterisation of the heterogenised aluminium isopropoxide over three supports (SiO_2_, TiO_2_ and γ-Al_2_O_3_). Whilst SiO_2_-based supports have been previously screened for heterogenisation of metal alkoxide species,^17,24,36,37^ the use of other supports remained unexplored. It is therefore of interest to test the general validity of metal alkoxide heterogenisation over supports for MPV reductions. The synthesised catalysts were initially evaluated for the reduction of propionaldehyde using 2-propanol as the hydride source. The results show a very good yield up to 90% and selectivity of 100% for Al(O^i^Pr)_3_-SiO_2_. Table S1 of the ESI† shows the catalytic yield and selectivity for other aldehydes and ketones over the heterogenised catalysts Al(O^i^Pr)_3_-SiO_2_, Al(O^i^Pr)_3_-TiO_2_ and Al(O^i^Pr)_3_-Al_2_O_3_. As observed from the data, the corresponding unsaturated alcohols were formed by reducing the unsaturated aldehydes and ketones with high selectivity and no other reduction products were found. A series of other carbonyl compounds was tested and generally good catalytic yields to the corresponding alcohol for cinnamaldehyde, benzaldehyde, cyclohexanone and acetophenone were obtained. After 3 hours, benzaldehyde was highly reduced to benzyl alcohol with a yield ranging between 87.7–95.7% across the three heterogenised catalysts. Out of the studied carbonyl compounds, acetophenone has the lowest yield (<50%) while cinnamaldehyde was converted into its corresponding alcohol with a 55.4% yield after 6 hours. The trend observed in reactivity across the carbonyl compounds could be due to steric hindrance effects. Smaller aldehyde molecules, such as propionaldehyde and benzaldehyde, could have more access to the catalyst active sites. Diffusion and mass transfer effects could also play a role in restricting more bulky molecules from reaching catalytic active sites.^14^

It is noted that the unmodified SiO_2_, TiO_2_ and γ-Al_2_O_3_ were tested and found to be totally inactive for the reduction reactions, demonstrating that the reaction is mainly catalysed by the Al(O^i^Pr)_3_ immobilised over the surface.

In terms of comparing activity across different supports used for the catalyst preparation, the activity of the catalysts is in the order Al(O^i^Pr)_3_-SiO_2_ > Al(O^i^Pr)_3_-Al_2_O_3_ > Al(O^i^Pr)_3_-TiO_2_ for all aldehydes and ketones, although differences are not very large. The activity of Al(O^i^Pr)_3_-SiO_2_ and Al(O^i^Pr)_3_-Al_2_O_3_ are comparable while Al(O^i^Pr)_3_-TiO_2_ gives lower yield values. This may be attributed to lower Al(O^i^Pr)_3_ catalyst loading observed in Al(O^i^Pr)_3_-TiO_2_ as suggested by ICP-OES data in Table 1. The effectiveness of MPV reduction reactions is known to be affected by Lewis acid sites of the metal centre and its ligand exchange abilities.^3,4^ However, these factors are significantly affected by the type of ligand and coordination geometry at the metal centre.^19^ Al species prepared by grafting method on siliceous materials are known to contain more acid aluminium centres compared to those prepared by other conventional methods.^38^ The presence of aluminium coordinated in the form of heavily distorted tetrahedrons is hypothesised to be responsible for the presence of more acidic aluminium centres, which enhance the catalytic activity of such materials.^19^ Another important advantage of the grafted catalyst compared to the homogeneous one is that the aluminium alkoxide molecules cannot self-assemble due to surface confinement, and the support material, for example silica, is known to serve as an electron-withdrawing medium.^19^

To compare the activity of the heterogenised catalysts with the homogeneous counterpart, the same amount of the aluminium isopropoxide (Al(O^i^Pr)_3_) catalyst found in the heterogenised catalysts was used for the reduction of the studied aldehydes and ketones in homogeneous phase. As shown in Table 2, the turnover frequency (TOF) of the heterogenised Al(O^i^Pr)_3_-SiO_2_ and Al(O^i^Pr)_3_-Al_2_O_3_ are very similar to the values reported for the homogeneous Al(O^i^Pr)_3_ for all aldehydes and ketones. The TOF values observed are comparable to values reported in literature for the MPV promoted by heterogeneous catalysts whereas the selectivity reported in our case is higher.^39^ Similar findings have been reported in the heterogenisation of boron alkoxides on MCM-41 support.^16^ The heterogeneous B(O^i^Pr)_3_-MCM-41 catalyst showed similar catalytic activity to that of homogeneous boron isopropoxide (B(O^i^Pr)_3_) and boron tri-ethoxide (B(OEt)_3_) catalysts.^24^ This finding is particularly worth highlighting as it is usually reported that when grafted over supports, homogeneous catalysts have a markedly reduced catalytic activity.^40^ In our case, the catalytic activity remains essentially unaltered after the homogeneous catalyst is immobilised, with possibility to achieve this over different supports, which suggests that it is possible to run such reactions effectively whilst being able to easily separate and recycle the catalyst. The excellent catalytic activity and high selectivity of the heterogenised catalysts could be attributed to the presence of well-defined, single-site catalytic centres over the solid support, which also minimise formation of byproducts.^41^ Constraint effects could also play a role in the high performance of the supported catalysts.^42,43^

**Table 2:** TOF values for MPV reduction of aldehydes and ketones using homogeneous and heterogenised catalysts^a^

Entry	Substrate	Catalyst	TOF (s^−1^)	TOF (h^−1^)
1	Propionaldehyde	Al(O^i^Pr)_3_	1.95 × 10^−4^	0.70
2	Propionaldehyde	Al(O^i^Pr)_3_-SiO_2_	1.84 × 10^−4^	0.66
3	Propionaldehyde	Al(O^i^Pr)_3_-TiO_2_	1.69 × 10^−4^	0.61
4	Propionaldehyde	Al(O^i^Pr)_3_-Al_2_O_3_	1.78 × 10^−4^	0.64
5	Cinnamaldehyde	Al(O^i^Pr)_3_	7.50 × 10^−5^	0.27
6	Cinnamaldehyde	Al(O^i^Pr)_3_-SiO_2_	7.33 × 10^−5^	0.26
7	Cinnamaldehyde	Al(O^i^Pr)_3_-TiO_2_	6.63 × 10^−5^	0.24
8	Cinnamaldehyde	Al(O^i^Pr)_3_-Al_2_O_3_	7.22 × 10^−5^	0.26
9	Benzaldehyde	Al(O^i^Pr)_3_	2.58 × 10^−4^	0.93
10	Benzaldehyde	Al(O^i^Pr)_3_-SiO_2_	2.53 × 10^−4^	0.91
11	Benzaldehyde	Al(O^i^Pr)_3_-TiO_2_	2.32 × 10^−4^	0.84
12	Benzaldehyde	Al(O^i^Pr)_3_-Al_2_O_3_	2.41 × 10^−4^	0.87
13	Cyclohexanone	Al(O^i^Pr)_3_	1.25 × 10^−4^	0.45
14	Cyclohexanone	Al(O^i^Pr)_3_-SiO_2_	1.23 × 10^−4^	0.44
15	Cyclohexanone	Al(O^i^Pr)_3_-TiO_2_	1.15 × 10^−4^	0.41
16	Cyclohexanone	Al(O^i^Pr)_3_-Al_2_O_3_	1.20 × 10^−4^	0.43
17	Acetophenone	Al(O^i^Pr)_3_	8.05 × 10^−5^	0.29
18	Acetophenone	Al(O^i^Pr)_3_-SiO_2_	7.86 × 10^−5^	0.28
19	Acetophenone	Al(O^i^Pr)_3_-TiO_2_	7.22 × 10^−5^	0.26
20	Acetophenone	Al(O^i^Pr)_3_-Al_2_O_3_	7.69 × 10^−5^	0.27

a TOF (s^−1^) was calculated using the expression: TOF = mmol_product_/(mmol_catalyst_ × time (s)).

### 3.3. Effect of support surface

Using the same amount of Al(O^i^Pr)_3_, the catalytic activity of the grafted catalyst on SiO_2_, TiO_2_, and γ-Al_2_O_3_ was evaluated. A high catalytic performance was recorded for all the three catalysts in the reaction. Compared to Al(O^i^Pr)_3_-SiO_2_ and Al(O^i^Pr)_3_-Al_2_O_3_ catalysts, the Al(O^i^Pr)_3_-TiO_2_ heterogeneous catalyst displays a lower yield. This could be due to the smaller surface area of the TiO_2_ support, which can affect the aluminium isopropoxide dispersion. It was observed that the Al(O^i^Pr)_3_-SiO_2_ catalyst had similar activity to Al(O^i^Pr)_3_-Al_2_O_3_ catalyst for MPV reduction of unsaturated aldehydes and ketones. These findings demonstrate that the slight differences in the structural characteristics of the two support materials, SiO_2_ and γ-Al_2_O_3_, have little impact on the catalytic activity, with Al(O^i^Pr)_3_-SiO_2_ showing slightly higher activity than Al(O^i^Pr)_3_-Al_2_O_3_. Mesoporous materials with a large surface area, such as SiO_2_ and γ-Al_2_O_3_, offer a better support for the heterogenisation of metal alkoxides, such as Al(O^i^Pr)_3_. For most liquid phase processes, it is generally required for the support to have a relatively high surface area (usually >100 m^2^ g^−1^) such that the active sites are well dispersed and easily accessible on the surface of the support.^44^ Additionally, appropriate pore size is required to allow easy diffusion of reactants to the active sites of the catalyst.^45^ As such, the pore structure of these support materials, SiO_2_ and γ-Al_2_O_3_, likely favours easier access of the substrate to the aluminium centres, thus facilitating the MPV reduction reaction.

### 3.4. Activity of reused catalysts

**Fig. 13:**
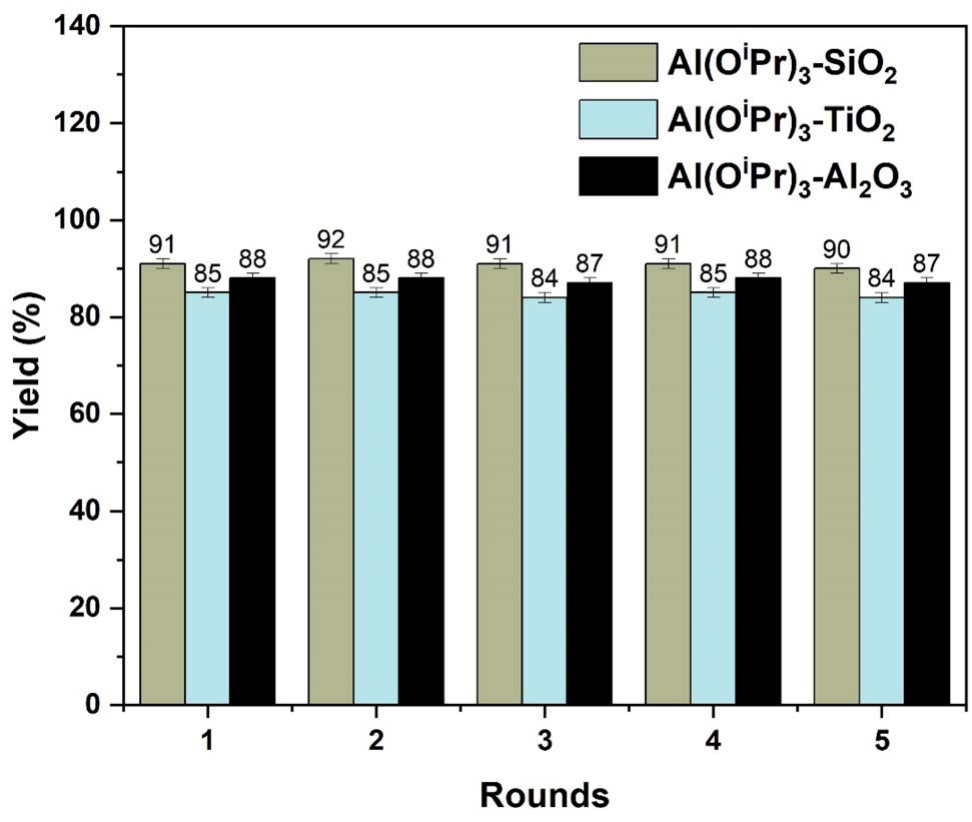
Recycling performance of Al(O^i^Pr)_3_-SiO_2_, Al(O^i^Pr)_3_-TiO_2_, and Al(O^i^Pr)_3_-Al_2_O_3_ for the MPV reduction of propionaldehyde.

After the grafted catalyst was refluxed in 2-propanol, the filtrate was found to be inactive for the MPV reduction of propionaldehyde, ruling out the possible presence of any leached materials in the reaction medium. The recovered catalyst was evaluated in a subsequent batch reaction after being cleaned with 2-propanol following each round of reaction. Fig. 13 shows the activity of the three supported catalysts after five rounds of reaction, using propionaldehyde as a model reaction for testing catalyst stability. The information in Fig. 13 shows that the activities of Al(O^i^Pr)_3_-SiO_2_, Al(O^i^Pr)_3_-TiO_2_, and Al(O^i^Pr)_3_-Al_2_O_3_ for the MPV reduction of propionaldehyde remain high even after 5 rounds of reaction and the selectivity to 1-propanol in each case was close to 100%. This demonstrates that the heterogenised catalysts have an exceptional stability.

XRD pattern of the recycled catalysts were obtained after 5 rounds of reuse as shown in Fig. 4(c), 5(c) and 6(c). The XRD pattern of all the three recycled catalysts (Al(O^i^Pr)_3_-SiO_2_, Al(O^i^Pr)_3_-TiO_2_, Al(O^i^Pr)_3_-Al_2_O_3_) maintained characteristic peaks similar to the initial substrates. This clearly indicates that even after rounds of reaction, the materials still retain their pore structure. The observed decrease in intensities of the peak may be due to plugging of the pores after several rounds of reuse.

## 4 Conclusions

We demonstrated that heterogenised catalysts synthesised by grafting of aluminium isopropoxide over mesoporous supports (SiO_2_, TiO_2_ and γ-Al_2_O_3_) are efficient and stable catalysts for the MPV reduction of various aldehydes and ketones. The use of mesoporous supports improves the dispersion the Al(O^i^Pr)_3_, hence resulting in high catalytic activity and selectivity of the catalysts. Higher catalytic activity was observed in Al(O^i^Pr)_3_-SiO_2_ compared to Al(O^i^Pr)_3_-TiO_2_, and Al(O^i^Pr)_3_-Al_2_O_3_. This may be due to differences in surface area and pore volume that could restrict the carbonyl compounds from accessing the catalyst active sites. All the heterogenised catalysts have an activity that is essentially the same of the homogeneous counterpart and show an excellent stability, displaying the ability to be recyclable up to 5 rounds of reaction with no significant decrease in activity. In summary, the work clearly shows that it is possible to adopt strategies of homogeneous catalyst immobilisation to perform chemical reactions, usually performed in homogeneous phase, using solid catalysts that are able to keep the same activity as the homogeneous counterpart and with excellent stability, which allows their ease of separation and reuse, hence avoiding waste of valuable catalytic materials. The approach adopted here could be further explored to develop strategies for more complex organic syntheses involving drug molecules, natural products or a wide range of substrates, including sterically hindered carbonyl compounds.

## Data availability

The data that support the findings of this study are available within the article and the ESI.†

## Conflicts of interest

There are no conflicts to declare.

## Supplementary Material

RA-012-D2RA06437E-s001
