# Supplementary material for: Activity and stability studies of H-transfer reduction reactions of aldehydes and ketones over aluminium isopropoxide heterogenised catalysts
Source: RSC Adv. 2022 Nov 29;12(52):33970–80. doi: 10.1039/d2ra06437e (PMC9706686; doi:10.1039/d2ra06437e)
Supplement: RA-012-D2RA06437E-s001 [file RA-012-D2RA06437E-s001.pdf]

Electronic Supplementary Material (ESI) for RSC Advances.
This journal is © The Royal Society of Chemistry 2022

Supporting Information

Activity and stability studies of H-transfer reduction reactions of aldehydes and ketones over aluminium isopropoxide heterogenised catalysts

Atika Muhammad[a], Ammaru Ismaila[a], Bashir Jelani Usman[b], Graziano Di Carmine[c], Carmine D'Agostino[a, d]*

[a]Department of Chemical Engineering, The University of Manchester, Oxford Road, M13 9PL, United Kingdom

[b]Department of Materials, The University of Manchester, Oxford Road, M13 9PL Manchester, United Kingdom

[c]Dipartimento di Scienze Chimiche, Farmaceutiche ed Agrarie, Università degli Studi di Ferrara, Via L. Borsari, 46, I-44121 Ferrara, Italy

[d]Dipartimento di Ingegneria Civile, Chimica, Ambientale e dei Materiali (DICAM), Alma Mater Studiorum – Università di Bologna, Via Terracini, 28, 40131 Bologna, Italy

*Corresponding Author:

Dr Carmine D'Agostino

Email: carmine.dagostino@manchester.ac.uk; carmine.dagostino@unibo.it

The yield was calculated using the following expression:

$$Y(\%) = \frac{moles\ of\ product}{initial\ moles\ of\ limiting\ reactant} \times 100$$

Equation S1

The turnover frequency (TOF) was determined using the expression:

$$TOF = \frac{mmol_{product}}{mmol_{catalyst} \times time(s)}$$

Equation S2

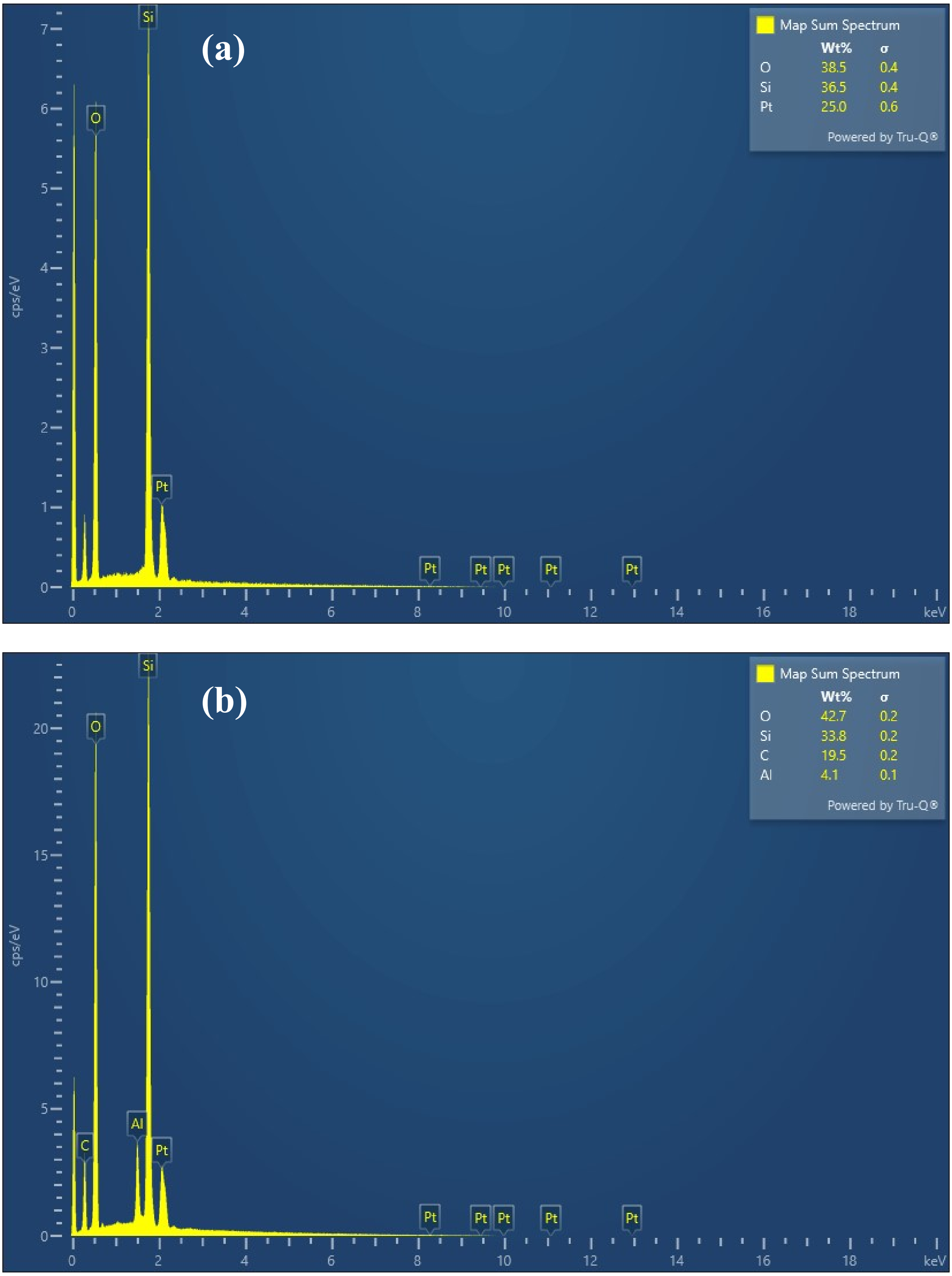

Figure S1. EDX spectrum of (a) SiO_2 and (b) $Al(O^iPr)_3$-SiO_2. Pt comes from the coating of the sample as part of sample preparation for the analysis.

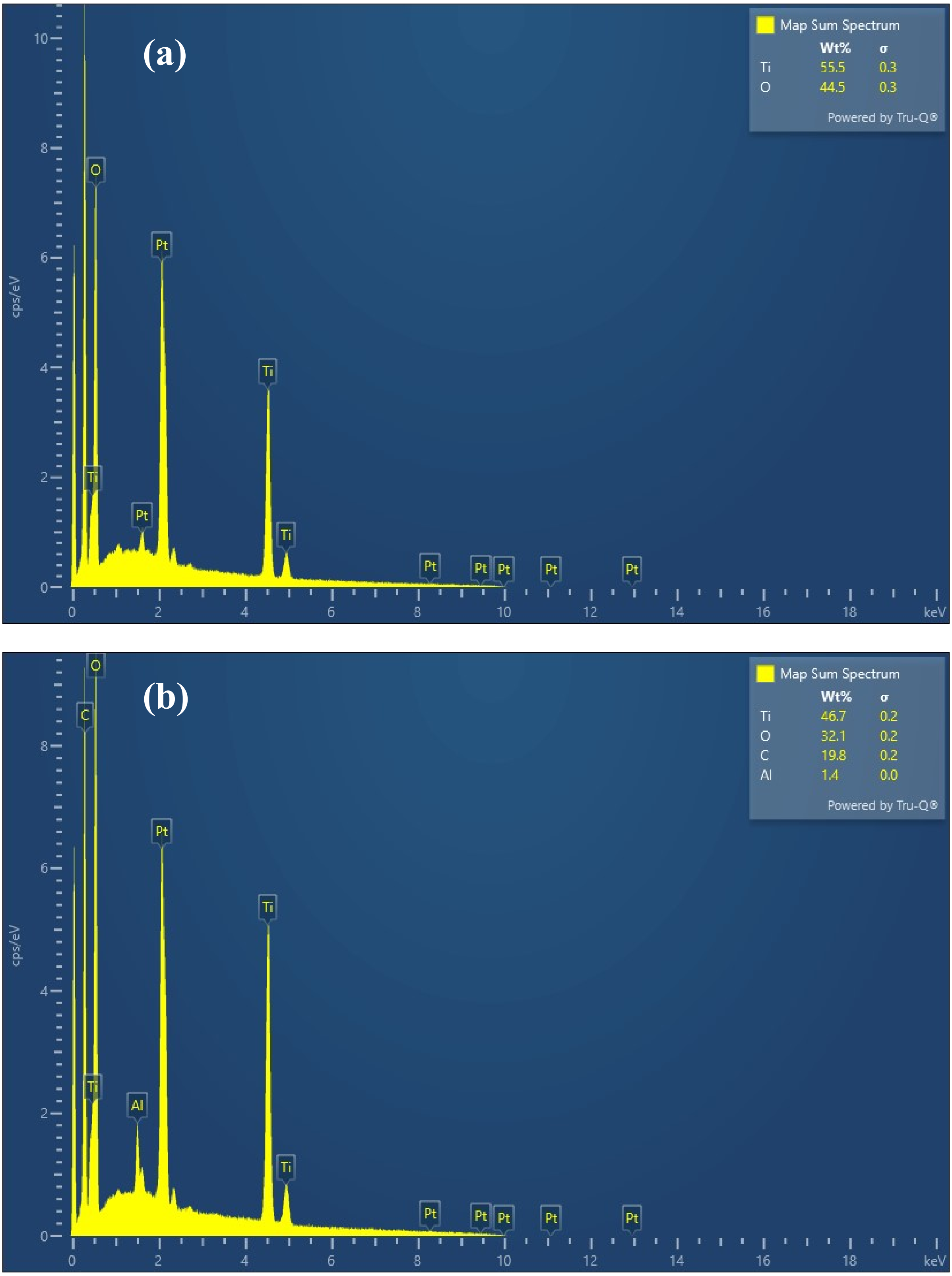

Figure S2. EDX spectrum of (a) TiO_2 and (b) $Al(O^iPr)_3$-TiO_2. Pt comes from the coating of the sample as part of sample preparation for the analysis.

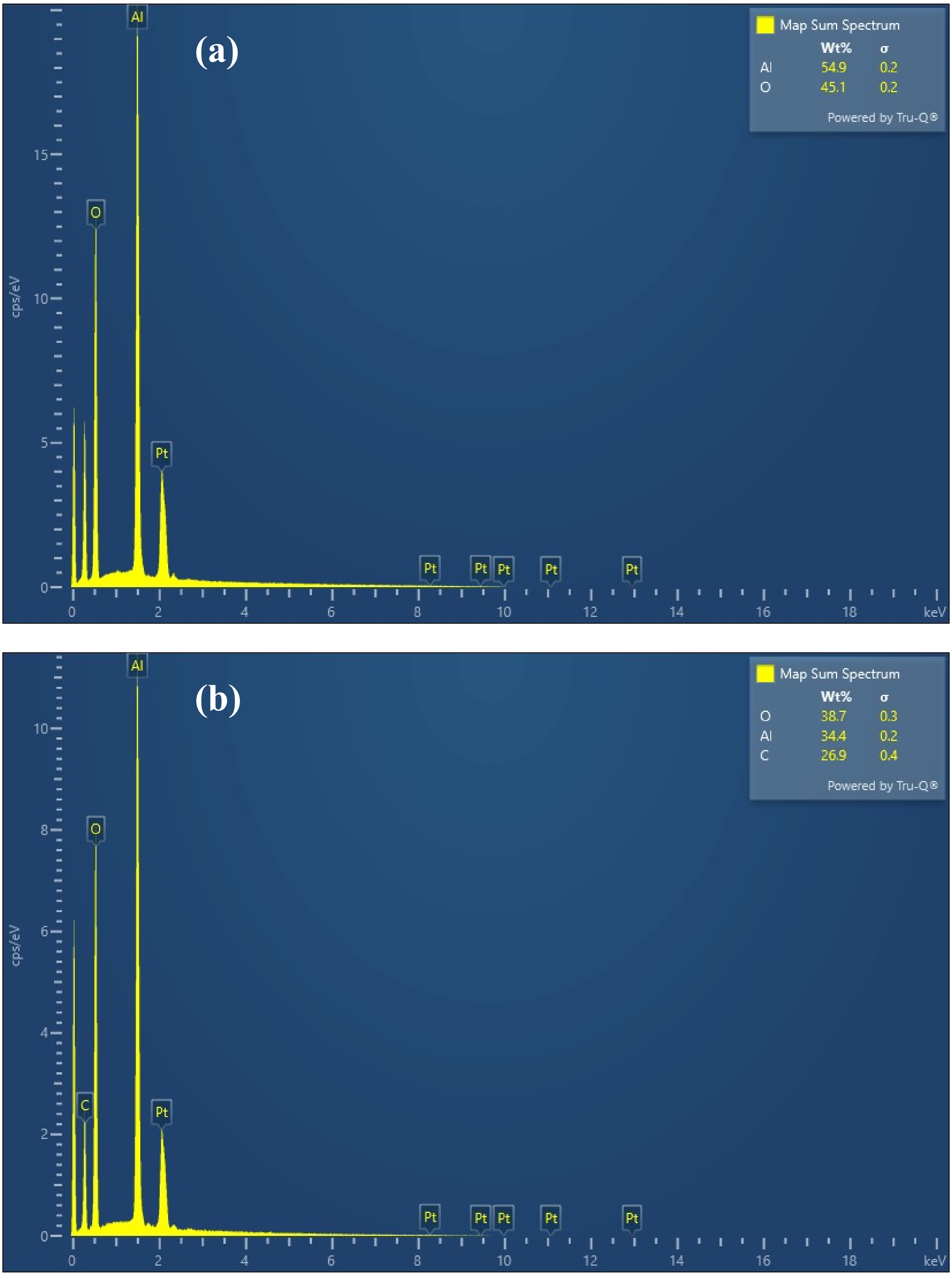

Figure S3. EDX spectrum of (a) Al_2O_3 and (b) $Al(O^iPr)_3$)-Al_2O_3. Pt comes from the coating of the sample as part of sample preparation for the analysis.

Table S1. Reaction data for the MPV reduction of aldehydes and ketones using heterogenised catalysts.

Substrate	$Al(O^iPr)_3$-SiO_2			$Al(O^iPr)_3$-TiO_2			$Al(O^iPr)_3$-Al_2O_3		
	Time (h)	Yield (%)	Selectivity (%)	Time (h)	Yield (%)	Selectivity (%)	Time (h)	Yield (%)	Selectivity (%)
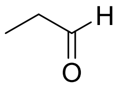 Propionaldehyde	4	92.5	100	4	85.2	100	4	89.9	100
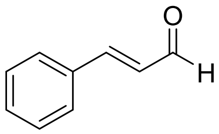 Cinnamaldehyde	4	55.4	100	6	50.1	100	6	54.6	100
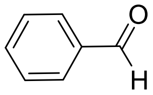 Benzaldehyde	3	95.7	100	3	87.7	100	3	91.2	100
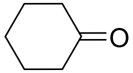 Cyclohexanone	5	77.5	100	5	72.2	100	5	75.6	100
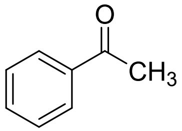 Acetophenone	5	49.5	100	5	45.5	100	5	48.4	100
